# A Comparative Study of 1-Year Postprocedural Outcomes in Transcatheter Mitral Valve Repair in Advanced Primary Mitral Regurgitation: PASCAL vs. MitraClip

**DOI:** 10.3390/jcm13020484

**Published:** 2024-01-16

**Authors:** Felix Rudolph, Johannes Kirchner, Maria Ivannikova, Vera Fortmeier, Tanja Katharina Rudolph, Kai Peter Friedrichs, Volker Rudolph, Muhammed Gerçek

**Affiliations:** Clinic for General and Interventional Cardiology/Angiology, Herz- und Diabeteszentrum NRW, Ruhr-Universität Bochum, 32545 Bad Oeynhausen, Germany; frudolph@hdz-nrw.de (F.R.);

**Keywords:** primary mitral regurgitation, advanced mitral regurgitation, MitraClip, PASCAL, transcatheter therapy, M-TEER

## Abstract

Both the MitraClip and PASCAL systems offer transcatheter edge-to-edge repair (TEER) solutions for mitral regurgitation. Evidence indicates a lower technical success rate for TEER in complex degenerative mitral regurgitation (DMR) cases. We conducted a retrospective analysis of patients who underwent transcatheter edge-to-edge therapy for primary mitral regurgitation with advanced anatomy, defined as mitral regurgitation effective regurgitant orifice area (MR-EROA) ≥0.40 cm^2^ or large flail gap (≥5 mm) or width (≥7 mm) or Barlow’s disease, that completed follow-up after 1 year. Our criteria were met by 27 patients treated with PASCAL and 18 with MitraClip. All patients exhibited a significant, equivalent short-term reduction in MR-EROA, mitral regurgitation vena contracta diameter (MR-VCD), regurgitant volume, and clinical status. At 1 year follow-up, reductions in MR-VCD, regurgitant volume, and MR-EROA remained significant for both groups without significant differences between groups. MR-Grade ≤ 1+ was achieved in 18 (66.7%) and 10 (55.6%) patients, respectively. At follow-up, no difference in hospitalization for cardiac decompensation was observed. Overall death was similar in both groups. Our study suggests that both the PASCAL and MitraClip systems significantly reduce mitral regurgitation even in advanced degenerative diseases. Within our limited data, we found no evidence of inferior performance of the PASCAL system.

## 1. Introduction

An untreated severe degenerative mitral regurgitation (DMR) is associated with high patient suffering and an unfavorable prognosis [1,2]. If possible, surgical repairs or replacements are the preferred therapeutical options [2], but with progressively older and more morbid patients, the perioperative risks are frequently deemed intolerable. Both the MitraClip (Abbott Laboratories, Chicago, IL, USA) and PASCAL (Edwards Lifesciences, Irvine, CA, USA) systems allow for a mitral transcatheter edge-to-edge repair (M-TEER), offering a less invasive approach [3]. Notably, the PASCAL system is newer and less established, especially in patients with advanced and complex anatomies, for whom recent data suggest a worse technical success rate in comparison to all-comer data [4,5]. Data from the CLASP IID registry revealed a noninferiority of the PASCAL device compared to the MitraClip system in patients with DMR at 30 days and 6 months follow-up [1,4]. Additionally, these data indicated that MR-Grade worsened 6 months after M-TEER compared with discharge with an allegedly more sustained MR reduction in the PASCAL group [4,5]. However, data with a longer follow-up time in patients with advanced mitral regurgitation due to complex anatomy remains scarce. In 2020, a comparison of acute procedural results and short-term follow-up data (30 days) presented similar efficacy and safety in patients with advanced DMR undergoing M-TEER with the PASCAL or the MitraClip system [6]. In the current study, we evaluated patients with advanced DMR who underwent either a PASCAL or MitraClip procedure at our clinic and completed follow-up after 1 year.

## 2. Materials and Methods

### 2.1. Study Design

**Figure 1:**
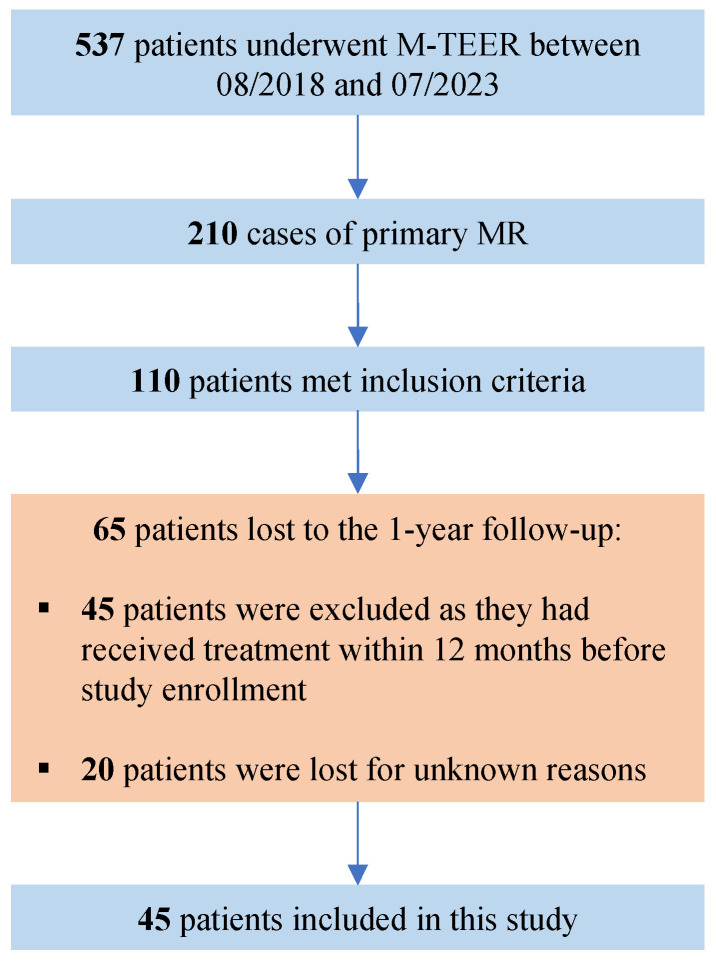
Flow-chart for patient selection. M-TEER = transcatheter edge-to-edge repair for mitral regurgitation, MR = mitral regurgitation.

In this retrospective single-center study, we conducted an analysis of all patients who underwent M-TEER for advanced mitral regurgitation at the Heart and Diabetes Center NRW of Ruhr University in Bochum, a high-volume center for M-TEER in Germany, until July 2023 (Figure 1). The null hypothesis asserts no significant difference in echocardiographic outcomes one year after M-TEER for severe mitral regurgitation in patients with advanced anatomies, comparing cases treated with the PASCAL system to those treated with MitraClip. The primary endpoint is MR-Grade at one-year follow-up, with secondary endpoints including changes in echocardiographic parameters such as mitral regurgitation effective regurgitant orifice area (MR-EROA), mitral regurgitation vena contracta diameter (MR-VCD), regurgitant volume, and PISA radius. As an additional secondary endpoint, we assessed mortality at study enrollment in July 2023 through independent queries to the German Registration Office, unrelated to clinical visits.

The main inclusion criteria were the presence of an advanced DMR (defined as MR-EROA ≥ 0.40 cm^2^ or large flail gap (≥5 mm) or width (≥7 mm) or Barlow’s disease) and a completed 1-year-follow-up to assess the postprocedural outcomes. The definition of advanced DMR was derived from a previous study conducted by our research group [6]. The current study aims to present long-term data within this uncommon patient cohort. The therapeutic approach (surgical vs. transcatheter) was decided for each patient individually by an interdisciplinary Heart Valve Team. The implanted device (PASCAL or MitraClip) was chosen by the interventionalist. The present study had no influence on patient or device selection. All interventions were performed by experienced interventional cardiologists with more than 300 performed cases. The study was approved by the local Ethics Committee of the Ruhr University of Bochum and carried out in accordance with the Declaration of Helsinki.

### 2.2. Echocardiographic Assessment

All patients underwent transesophageal and transthoracic echocardiography before intervention (Vivid E95, General Electric Healthcare, Chicago, IL, USA). At follow-up, a transthoracic echocardiography was performed. Acquired images were stored digitally in accordance with the German Data Protection Regulations. The echocardiographic parameters of the selected patients were evaluated in a retrospective manner (EchoPac Version 203 (Revision 66.0) on the Vivid E95 system by General Electric Healthcare, Chicago, IL, USA). The measurements of MR-VCD and PISA radius were adjusted to a Nyquist limit of 30–40 cm/s. To assess the interobserver reliability (IOR) of the measurements, a randomized subset of 50% was analyzed by two independent investigators.

### 2.3. Statistical Analysis

Statistical analysis was performed using R in RStudio (Version 2023.06, RStudio Inc., Boston, MA, USA). Continuous variables were reported as mean ± standard deviation if normally distributed and in median and interquartile range (IQR) if not normally distributed. To determine if the data was normally distributed or not, the Shapiro–Wilk test was used. Categorial Variables were presented as frequencies and percentages. Group comparisons were calculated using the Wilcox test or Student’s *t*-test, respectively, for continuous variables. For binary data, the Chi-Square test was used after generating a contingency table. A categorial group comparison was calculated using Fisher’s exact test. A *p*-value < 0.05 was considered statistically significant. The interobserver reliability was calculated using the “irr” package (Version 0.84.1) in R. An IOR ≥ 0.7 was considered acceptable. Missing echocardiographic valuables were imputed using the “mice” package (Version 3.16.0) in R using Predictive Mean Matching.

## 3. Results

### 3.1. Study Population

In total, 537 patients underwent M-TEER at our clinic between August 2018 and July 2023. DMR was the main pathology in 210 of these patients. For DMR patients, age was similar between the groups (PASCAL: 83 (80–85) years; MitraClip: 83 (78–86) years), with no significant difference observed (*p* = 0.96). Other parameters, such as gender distribution, BMI, and prevalence of comorbidities, also exhibited no statistically significant disparities. Notable differences included a higher prevalence of COPD in the MitraClip group (PASCAL: 6.4%; MitraClip: 15.9%, *p* = 0.043), variations in fluoroscopy time, and disparities in certain mitral valve pathologies, such as flail leaflets. The baseline characteristics of all DMR patients are presented in Table 1.

The echocardiographic criteria for inclusion were matched by 110 of these patients at baseline, but data for 1-year follow-up was only available in 45 patients, of whom, 27 received treatment with PASCAL and 18 underwent MitraClip intervention. Out of the 38 patients from our previous study [6], 28 are also included in this study. Ten patients from the original study could not be included due to a lack of one-year follow-up. Seventeen patients are new and were not considered in the original study. Detailed characteristics of the advanced DMR patients are given in Table 2. The median age at intervention was comparable between both advanced DMR groups with 81.0 (78–85) years in the PASCAL group and 83 (77.0–86.8) years in the MitraClip group. The rate of female gender was higher in the MitraClip cohort (PASCAL: 25.9%; MitraClip: 38.9%), but not statistically significant. Regarding the recorded cardiovascular risk profile and comorbidities, no statistically significant difference was observed between the groups.

Parameters surrogating the clinical condition before intervention were also comparable between the groups. Measured levels of NT-proBNP were 2410.0 (892.0–3580.0) pg/mL in the patients treated with PASCAL and 3355.0 (1048.0–5690.0) pg/mL in the MitraClip cohort (*p* = 0.27). The Six-Minute-Walking Distance was not different between the groups (PASCAL: 270.0 ± 112.8 m; MitraClip 249.2 ± 118.8 m, *p* = 0.65). Calculated risk scores for cardiac surgery were similar in both groups.

### 3.2. Periprocedural Data

Technical success, according to the MVARC criteria, was achieved in 98.1% of all treated DMR patients. For advanced DMR patients, this was achieved in 100% of cases. In both advanced DMR groups, a median of 2 (1–2) devices were implanted (*p* = 0.77). Detailed numbers are presented in Table 3. Procedure times were similar. The fluoroscopy time was significantly lower in the PASCAL cohort (PASCAL: 6.8 (5.2–9.3) min; MitraClip: 8.8 (7.1–14.2) min, *p* = 0.026).

### 3.3. Baseline Echocardiography

For the advanced DMR groups, one patient (2.2%) was included due to a diagnosed Barlow’s disease. In total, 15 patients presented with a prolapse without a significant difference between the sub-cohorts (PASCAL: 11 (40.7%); MitraClip: 4 (22.2%), *p* = 0.33). In 29 patients, a flail was observed (PASCAL: 19 (70.4%); MitraClip: 10 (55.6%), *p* = 0.35). The flail gap (PASCAL: 4.0 (2.0–5.0) mm; MitraClip: 5.5 (2.3–6.8) mm, *p* = 0.29) and flail width (PASCAL: 9.7 ± 4.5 mm; MitraClip: 10.0 ± 3.1 mm, *p* = 0.83) were comparable. The baseline echocardiographic parameters are presented in Table 4.

All patients suffered from an MR-Grade ≥ 3+ before intervention. The MR-EROA (PASCAL: 0.6 (0.5–0.7) cm^2^; MitraClip: 0.5 (0.4–0.8) cm^2^, *p* = 0.47), MR-VCD (PASCAL: 9.7 ± 2.8 mm; MitraClip: 9.9 ± 2.5 mm, *p* = 0.78) and regurgitant volume (PASCAL: 87.5 ± 36.3 mL; MitraClip: 75.0 ± 37.4 mL, *p* = 0.29) were similarly distributed between the groups.

The interobserver reliability for the inclusion parameters at baseline showed satisfactory results (MR-EROA: 0.77; Flail gap: 0.82; Flail width: 0.84).

**Figure 2:**
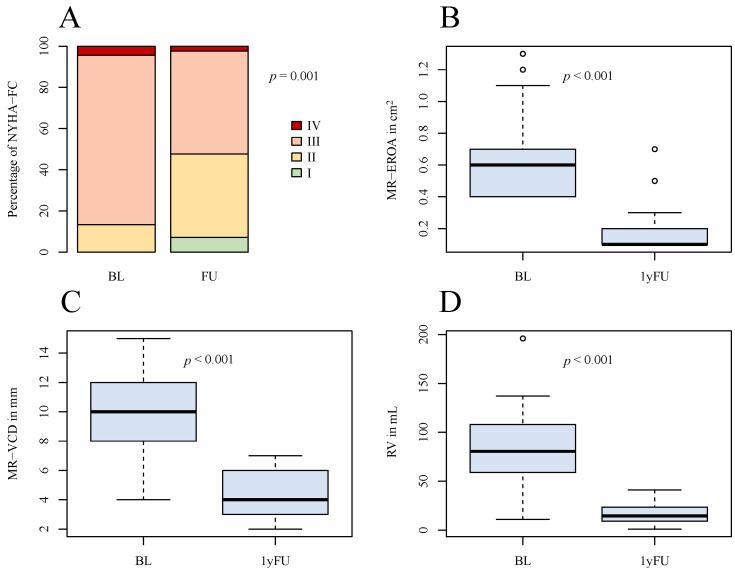
Graphical comparison of overall outcome after 1 year. (**A**) NYHA-FC at baseline and at follow-up; (**B**) boxplots of MR-EROA at baseline and at follow-up; (**C**) boxplots of MR-VCD at baseline and at follow-up; (**D**) boxplots of RV at baseline and at follow-up. NYHA-FC = NYHA functional class, MR-EROA = mitral regurgitant effective regurgitant orifice area, MR-VCD = mitral regurgitant vena contracta, RV = regurgitant volume, BL = baseline, 1yFU= follow-up after 1 year.

In all advanced DMR patients, a significant reduction of MR-EROA, MR-VCD, and regurgitant volume was observed with significant improvement of the clinical status. Prior to treatment, 39 patients (86.7%) were categorized with NYHA-FC ≥ III, while post-intervention, this number was reduced to 22 individuals (48.9%) (*p* < 0.01) (Figure 2). The Six-Minute-Walking-Distance increased from 240.0 [200.0–340.0] m to 320.0 [280.0–395.0] m (*p* < 0.01), and the levels of NT-proBNP had decreased from 2540.0 [743.0–4585.0] pg/mL to 1240.0 [465.0–2695.0] pg/mL (*p* = 0.026).

**Figure 3:**
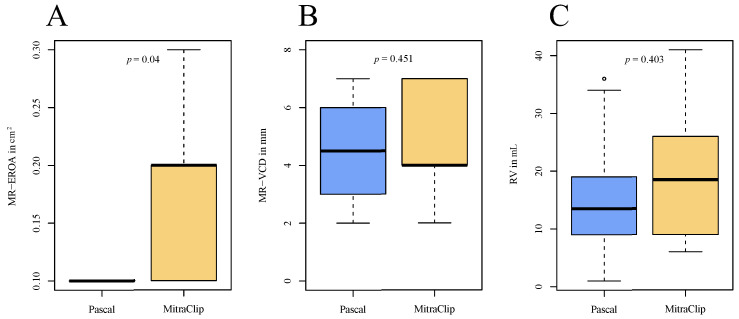
Comparison of echocardiographic parameters at 1-year follow-up between the advanced DMR groups. (**A**) Boxplots of MR-EROA at follow-up; (**B**) boxplots of MR-VDC at follow-up; (**C**) boxplots of RV at follow-up. MR-EROA = mitral regurgitant effective regurgitant orifice area, MR-VCD = mitral regurgitant vena contracta, RV = regurgitant volume.

At 1-year follow-up, MR-VCD, the regurgitant volume and PISA radius with their respective delta and the ΔMR-EROA showed no significant difference between the groups. In the advanced DMR cohorts, the transmitral gradient increased significantly from 2 (1–3) to 3 (2.4–4) mmHg (*p* < 0.01), with a lower delta increase observed in the MitraClip patients at follow-up (PASCAL: 1.9 ± 1.7 mmHg, MitraClip: 0.6 ± 1.6 mmHg; *p* = 0.028). The MR-EROA at 1-year follow-up was numerically lower in the PASCAL group as compared to the MitraClip group (0.1 (0.1–0.1) cm^2^ vs. 0.2 (0.1–0.2) cm^2^; *p* = 0.04; Figure 3 and Table 5).

**Figure 4:**
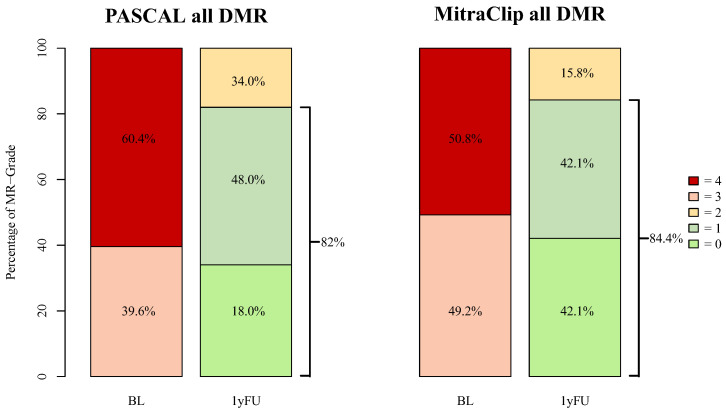
MR-Grades after PASCAL and MitraClip intervention for all treated DMR patients at baseline and 1-year follow-up. BL = baseline, 1yFU= follow-up after 1 year, 0–4 = MR-Grades 0–4, DMR = degenerative mitral regurgitation. Follow-up data were available in 42 (29.8%) of all DMR PASCAL and in 14 (20.3%) MitraClip patients.

**Figure 5:**
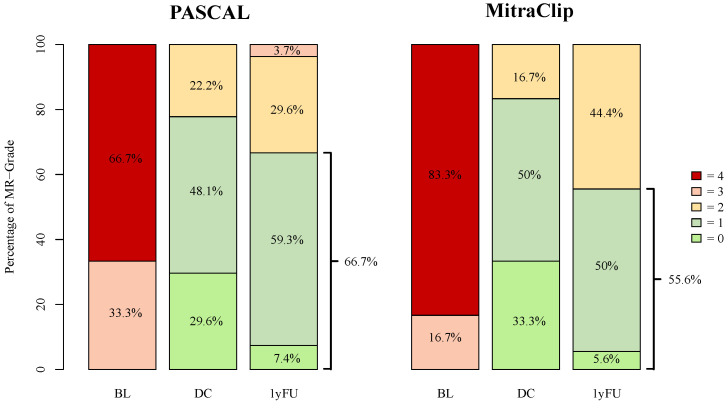
MR-Grades after PASCAL and MitraClip intervention for advanced DMR at baseline, discharge, and 1-year follow-up. BL = baseline, DC = discharge, 1yFU = follow-up after 1 year, 0–4 = MR-Grades 0–4.

For all DMR patients, the 1-year follow-up data were only available in 69 (32.9%) cases (PASCAL: 42 (29.8%); MitraClip: 14 (20.3%)). At one year follow-up, MR-Grade ≤ 1+ was observed in 82% of PASCAL and in 84.2% of MitraClip patients (Figure 4). At discharge, a reduction in MR-Grade ≤ 2+ was achieved in all advanced DMR patients. MR-Grade ≤ 1+ was achieved in 21 (77.8%) of PASCAL and in 15 (83.3%) of MitraClip patients. At 1-year follow-up, 26 (96.3%) patients in the advanced DMR PASCAL group and 18 (100.0%) of the MitraClip patients presented with an MR-Grade ≤ 2+, whereas a reduction in MR-Grade ≤ 1+ was achieved in 18 (66.7%) and 10 (55.6%), respectively (*p* = 0.88) (Figure 5). The decrease in the percentage of MR-Grade ≤ 1+ was not statistically significant between the advanced DMR groups (PASCAL: *p* = 0.45; MitraClip: *p* = 0.28).

### 3.4. Outcome

**Figure 6:**
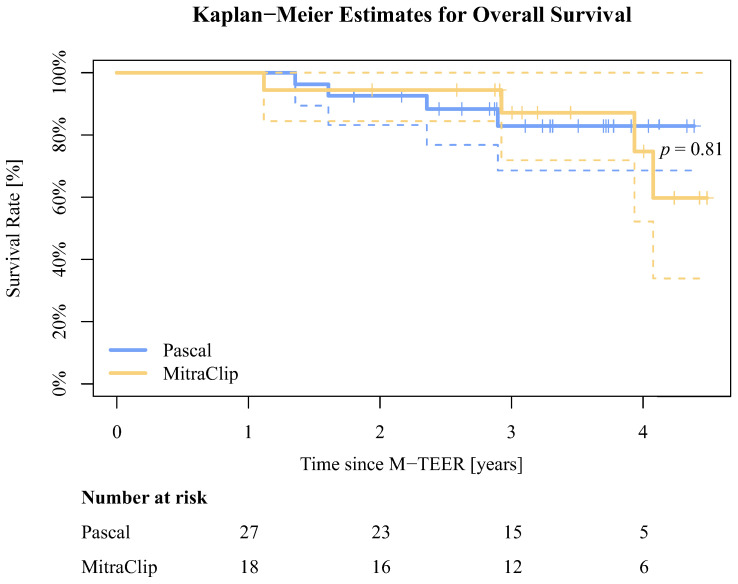
Kaplan–Meier estimates for overall survival for the advanced DMR groups. The dotted lines illustrate the confidence intervals.

Data for death status, acquired via query at the German Registration Office independently from clinical visits, were available for 196 out of all 210 (93.3%) DMR patients, and in 45 of 45 (100%) advanced DMR patients. The mean time to death was 487.8 ± 460.9 days for the group of all treated DMR patients. Of these patients, 42 (21,4%) had died in the PASCAL and 19 (28.8%) in the MitraClip group. Between discharge and 1-year follow-up, 5 (14.8%) patients in the advanced DMR PASCAL group had been hospitalized due to cardiac decompensation, and 3 (17.6%) in the MitraClip group (*p* > 0.99). The mean time to death was 1134.7 ± 316.9 days in the PASCAL group and 1213.9 ± 332.4 days in the MitraClip group. In total, 4 (14.3%) patients had died from overall cause in the PASCAL and 4 (23.5%) in the MitraClip group (*p* = 0.81). The Kaplan–Meier estimates for overall survival in the advanced DMR groups are presented in Figure 6.

## 4. Discussion

Our study further supports existing data that patients with advanced DMR, both the PASCAL and MitraClip systems have a robust safety profile and show favorable echocardiographic and clinical results one year after implantation. Within this study, we could not find evidence suggesting an inferiority of the PASCAL system compared to MitraClip. As depicted in Figure 1, advanced or complex DMR constitutes a rare subgroup among M-TEER patients, even in high-volume centers, and long-term data for this subgroup remains exceptionally scarce.

Currently, no anatomical characteristics conclusively favor one device over the other. In their respective current generations, the MitraClip offers four different sizes (NT/NTW and XT/XTW), while the PASCAL system provides two (ACE and P10). Theoretically, the MitraClip system allows for more individual adjustment to anatomical conditions due to its wider selection of clip sizes. In real-world experience, this advantage is counterbalanced by the greater variety of working catheters (three compared to two) in the PASCAL system, enabling a wider range of motion and ease of use in the left atrium. Additionally, the PASCAL system is equipped with a central spacer designed to reduce force on the leaflets.

The recent technical advancements in the latest iterations of both devices have already demonstrated their potential to enhance the M-TEER treatment of advanced and complex mitral anatomies. While it is anticipated that a learning curve will accompany these new device generations, it is noteworthy that interventionalists have already accumulated significant experience in M-TEER with previous device generations. Consequently, the impact of the learning curve on patient outcomes may only be partially evident. Besides improvements in M-TEER technologies, transcatheter mitral valve replacements (TMVR) have evolved as an appealing alternative interventional strategy for mitral regurgitation. However, its efficacy and safety, especially in high-risk patients, must still be proven. At present, Abbott’s TENDYNE is the only CE-certified TMVR device, which is accompanied by a more invasive transapical approach. To optimize the future selection of the most suitable therapeutic approach for individual patients, a better understanding of clinical and anatomical features will prove crucial. Our study population herein comprised patients with advanced degenerative mitral regurgitation, reflected by 63.0% having a flail leaflet as the underlying pathology. Before the intervention, the median of MR-VCD was 10 mm and MR-EROA was 0.6 cm^2^, which was previously shown to be associated with an increased risk for periprocedural failure [7]. In contrast, in the recently published data from the EXPAND G4 registry reported an MR-EROA of 0.338 ± 0.15 cm^2^ to be indicative of a “risk of inadequate mitral regurgitation reduction” cohort [8].

The CLASP IID registry defined complex anatomy as either ≥2 independent jets, severe bi-leaflet or multi-scallop prolapse, and mitral valve orifice area < 4.0 cm^2^ or flail gap > 10 mm or flail width > 10 mm, and, therefore, is broader than the definition we applied [4]. Certainly, CLASP IID will shortly provide deeper insight and longer follow-up data, given its relatively strict eligibility criteria and enrollment oversight by a screening committee. While our patients were not randomized and our study population is limited, our data reflect the current real-life situation for advanced DMR.

As Buzzatti et al. have previously shown, a reduction to a residual MR-Grade ≤ 1+ is associated with better outcomes compared to residual grade ≤ 2+ [9]. In our findings, the PASCAL system achieved a reduction of MR-Grade ≤ 1+ in 66.7% 1 year after intervention, compared to 55.6% in the MitraClip cohort. Our findings are in line with Hausleiter et al., who reported an MR-Grade ≤ 1+ in 66.7% at discharge and 56.1% 6 months after intervention [4].

We also observed a non-significant decrease in MR-Grade ≤ 1+ from discharge to follow-up. However, comparing our 1-year follow-up data with the echocardiographic results 6 months after the intervention as described for the CLASP IID registry, our data show no evidence of a continued decline in MR-Grade over time following M-TEER.

In all patients, a significant reduction of MR-EROA was observed. Interestingly, there was a numerically greater reduction in the PASCAL group. It is, however, noteworthy that the estimation of MR-EROA post-TEER is technically challenging due to multiple orifices and vena contracta diameters, resulting in a wider interobserver variance. More accurate results could be achieved by estimating the 3D vena contracta or vena contracta area, which, unfortunately, is currently not realistically achievable in clinical routine. With further advancements in echo technology and its increasing availability in the clinical setting, this method holds the promise of achieving more accurate and comparable results in the future.

While our primary focus in this study was on the impact of M-TEER within a one-year timeframe in advanced DMR patients, we acknowledge the inclusion of a survival analysis spanning multiple years in Figure 6. This deviation from the original study objective arose due to the indirect inclusion criterion of survival until the 1-year follow-up, marked by the completion of echocardiographic assessments for all patients. The Kaplan–Meier estimates show a statistically non-significant trend for lower all-cause mortality in the PASCAL group. This could, however, be attributed to a potential bias resulting from non-random device selection. It is possible that the use of the established MitraClip was more prevalent among patients with complex and severe medical conditions, which our statistical analysis may not adequately capture or represent. The PASCAL cohort of this study partially comprises patients from the early experience with the device at our clinic. Interestingly, the mean time to death appears to be longer in the advanced DMR groups compared to all treated DMR patients. Noting the limited significance of our sample size, this still indicates successful long-term treatment, even in advanced or complex anatomical conditions using M-TEER.

## 5. Limitations

Only advanced DMR patients who completed follow-up after 1 year were retrospectively included in this study. Follow-up data for all DMR patients were limited. Therefore, the cohorts exhibited a restricted sample size, further emphasizing the scarcity of long-term real-world data within this subgroup of advanced or complex DMR. The implanted device was selected at the discretion of the interventional cardiologist independently from this study. Our findings may, therefore, only serve as a basis for formulating hypotheses.

## 6. Conclusions

Our study further supports existing data that both the PASCAL and MitraClip systems can greatly reduce mitral regurgitation and, consequently, improve the quality of life in patients with advanced anatomies. We did not observe an inferiority of the PASCAL system in the defined endpoints of this study, which included PASCAL patients at an early stage of our experience with the system. Taking this into account, and with the advancing development of devices and the growing experience of the implanters, an increasing number of patients, including those with challenging anatomic conditions, will be suitable for M-TEER. For definitive conclusions, however, randomized, multi-center studies with larger study populations, such as from the CLASP IID registry, will be needed.

## Figures and Tables

**Table 1:** Baseline characteristics for all DMR patients.

		PASCAL	MitraClip	
	Availability	n = 141	n = 69	*p*
Age Median (IQR), years	210/210	83 (80–85)	83 (78–86)	0.96
Female Gender n (%)	210/210	78 (100)	40 (100)	0.77
BMI Median (IQR), kg/m^2^	210/210	24.7 (22.1–28.7)	24.6 (22.2–29.1)	0.89
Atrial Fibrillation n (%)	208/210	128 (91.4)	61 (89.7)	0.63
Arterial Hypertension n (%)	210/210	106 (75.2)	53 (76.8)	0.87
Diabetes n (%)	209/210	20 (14.3)	13 (18.8)	0.42
COPD n (%)	210/210	9 (6.4)	11 (15.9)	0.043
Coronary Artery Disease n (%)	210/210	63 (44.7)	36 (52.2)	0.38
LVEF Median (IQR), %	192/210	44 (38–51)	43 (36.5–48)	0.36
STS-Score Median (IQR), %	129/210	2.34 (1.6–3.4)	3.07 (1.5–5.9)	0.19
EuroSCORE II Median (IQR), %	171/210	3.63 (2.5–5.7)	4.65 (2.6–7.3)	0.15
GFR Median (IQR), mL/min	144/210	56 (41–71)	52 (33–72)	0.21
Creatinine Median (IQR), mg/dL	207/210	1.1 (0.8–1.4)	1.2 (1–1.7)	0.021
NT-proBNP Median (IQR), pg/mL	154/210	2010 (876.3–3985)	2910 (859.8–5807.5)	0.43
6-MWD mean ± SD, m	79/210	280 (160–360)	200 (180–250)	0.24
NYHA Grade	209/210	I:0; II:20; III:109; IV:12	I:0; II:6; III:52; IV:10	0.10
n of Clips Implanted n	209/210	1:59; 2:64; 3:15	1:35; 2:25; 3:8	0.10
Procedure Time Median (IQR), min	209/210	91 (66–115)	84 (66–114)	0.49
Fluoroscopy Time Median (IQR), min	143/210	7.1 (5.1–12.38)	10.2 (7–15.7)	0.020
Prolapse n (%)	141/210	69 (66.99)	28 (73.68)	0.30
Flail n (%)	141/210	53 (51.46)	13 (34.21)	0.007
Flail Gap Median (IQR), mm	72/210	4 (3–5)	3 (2–3)	0.16
Flail Width mean ± SD, mm	72/210	7 (5–9)	7 (5–8)	0.81
MR-EROA Median (IQR), cm^2^	190/210	0.4 (0.3–0.7)	0.4 (0.2–0.6)	0.21
Mean MV-Gradient Median (IQR), mmHg	186/210	2.4 (1.8–3.6)	2 (1.6–3)	0.14
MR-Grade n, Grade	204/210	I:1; II:0; III:54; IV:84	I:1; II:0; III:31; IV:33	0.19
TR-Grade n, Grade	196/210	0:13; I:55; II:33; III:25; IV:4; V:4	0:4; I:27; II:22; III:9; IV:0; V:0	0.66

**Table 2:** Baseline characteristics for the advanced DMR patients.

		PASCAL	MitraClip	
	Availability	n = 27	n = 18	*p*
Age Median (IQR), years	45/45	81 (78–85)	83 (77–86.75)	>0.99
Female Gender n (%)	45/45	7 (25.93)	7 (38.89)	0.51
BMI Median (IQR), kg/m^2^	45/45	25 (23.91–26.79)	24.46 (22.88–26.49)	0.43
Atrial Fibrillation n (%)	45/45	18 (66.67)	10 (55.56)	0.54
Arterial Hypertension n (%)	45/45	26 (96.3)	14 (77.78)	0.14
Diabetes n (%)	45/45	4 (14.81)	2 (11.11)	>0.99
COPD n (%)	45/45	4 (14.81)	3 (16.67)	>0.99
Coronary Artery Disease n (%)	45/45	10 (37.04)	7 (38.89)	>0.99
LVEF Median (IQR), %	45/45	55 (54.5–55)	55 (51.25–55)	0.60
STS-Score Median (IQR), %	45/45	2.07 (1.14–3.3)	2.28 (1.2–3.9)	0.69
EuroSCORE II Median (IQR), %	45/45	3.25 (2.07–4.98)	3.62 (2.79–4.6)	0.35
GFR Median (IQR), mL/min	45/45	58 (39–70.5)	51 (35–71.75)	0.67
Creatinine Median (IQR), mg/dL	45/45	1.1 (0.95–1.4)	1.05 (0.94–1.67)	0.74
NT-proBNP Median (IQR), pg/mL	39/45	2410 (892–3580)	3355 (1048–5690)	0.27
6-MWD mean ± SD, m	27/45	270 ± 112.8	249.23 ± 118.78	0.65
NYHA Grade	45/45	I:0; II:3; III:23; IV:1	I:0; II:3; III:14; IV:1	0.77

**Table 3:** Periprocedural data.

		PASCAL	MitraClip	
	Availability	n = 27	n = 18	*p*
n of Clips Implanted n	45/45	1:9; 2:16; 3:2	1:7; 2:8; 3:3	0.77
Type of Clips n	45/45	P10: 22; ACE: 6	XTR: 16; NTR: 4; NT: 1; NTW: 1	-
Procedure Time Median (IQR), min	45/45	103 (82–123.5)	88 (72.75–115.5)	0.52
Fluoroscopy Time Median (IQR), min	45/45	6.8 (5.15–9.25)	8.75 (7.05–14.23)	0.026
Dose Area Product Median (IQR), min	45/45	374 (212.05–680.95)	324 (224.25–685.15)	0.72

**Table 4:** Echocardiographic data for the advanced DMR patients at baseline.

		PASCAL	MitraClip	
	Availability	n = 27	n = 18	*p*
M. Barlow n (%)	45/45	0 (0)	1 (5.56)	0.40
Prolapse n (%)	45/45	11 (40.74)	4 (22.22)	0.33
Flail n (%)	45/45	19 (70.37)	10 (55.56)	0.35
Flail Gap Median (IQR), mm	29/45	4 (2–5)	5.5 (2.25–6.75)	0.29
Flail Width mean ± SD, mm	29/45	9.68 ± 4.46	10 ± 3.13	0.83
MR-EROA Median (IQR), cm^2^	43/45	0.6 (0.45–0.7)	0.45 (0.4–0.8)	0.47
Regurgitant Volume Median (IQR), mL	42/45	87.54 ± 36.27	75 ± 37.36	0.29
MR-VCD Median (IQR), mm	45/45	9.67 ± 2.81	9.89 ± 2.47	0.78
PISA radius Median (IQR), mm	45/45	9.33 ± 2.06	10.39 ± 2.83	0.18
Mean MV-Gradient Median (IQR), mmHg	38/45	2 (1–3)	2.5 (2–3)	0.20
MR-Grade n, Grade	45/45	I:0; II:0; III:9; IV:18	I:0; II:0; III:3; IV:15	0.23

**Table 5:** Echocardiographic data at follow-up.

		PASCAL	MitraClip	
	Availability	n = 27	n = 18	*p*
MR-EROA Median (IQR), cm^2^	44/45	0.1 (0.1–0.1)	0.2 (0.1–0.2)	0.04
ΔMR-EROA Median (IQR), cm^2^	42/45	0.5 (0.4–0.6)	0.3 (0.3–0.6)	0.18
Regurgitant Volume mean ± SD, mL	44/45	13.5 (9.3–18.5)	18.5 (9.3–26)	0.4
ΔRegurgitant Volume mean ± SD, mL	41/45	70.7 ± 34.9	56.8 ± 38	0.19
MR-VCD median (IQR), mm	44/45	4.5 (3–6)	4 (4–6.75)	0.45
ΔMR-VCD median (IQR), mm	44/45	5.2 ± 3.2	5 ± 2.9	0.81
PISA Radius median (IQR), mm	44/45	5 (4–5)	5 (4–6)	0.29
ΔPISA Radius mean ± SD, mm	44/45	4.5 ± 2.8	5.2 ± 3.1	0.47
Mean MV-Gradient Median (IQR), mmHg	39/45	3 (2.9–4.5)	3 (1.8–4)	0.22
ΔMean MV-Gradient Median (IQR), mmHg	36/45	1.9 ± 1.7	0.6 ± 1.6	0.028
MR-Grade n, Grade	45/45	I:16; II:8; III:1; IV:0	I:9; II:8; III:0; IV:0	0.54
ΔMR-Grade median (IQR), Grade	45/45	2 (2–3)	2 (2–3)	0.87

## Data Availability

The raw data supporting the conclusions of this article will be made available by the authors on request.
